# Supplementary material for: Realization of a two-dimensional Weyl semimetal and topological Fermi strings
Source: Nat Commun. 2024 Jul 17;15:6001. doi: 10.1038/s41467-024-50329-6 (PMC11255256; doi:10.1038/s41467-024-50329-6)
Supplement: Supplementary file 1 — Supplementary Information [file 41467_2024_50329_MOESM1_ESM.pdf]

Supplementary Information: Realization of a two-dimensional Weyl semimetal and topological Fermi strings

Qiangsheng Lu, P. V. Sreenivasa Reddy, Hoyeon Jeon, Alessandro R. Mazza, Matthew Brahlek, Weikang Wu, Shengyuan A. Yang, Jacob Cook, Clayton Conner, Xiaoqian Zhang, Amarnath Chakraborty, Yueh-Ting Yao, Hung-Ju Tien, Chun-Han Tseng, Po-Yuan Yang, Shang-Wei Lien, Hsin Lin, Tai-Chang Chiang, Giovanni Vignale, An-Ping Li*, Tay-Rong Chang*, Rob G. Moore*, Guang Bian*

E-mail: apli@ornl.gov,u32trc00@phys.ncku.edu.tw,moorerg@ornl.gov,biang@missouri.edu

Contents

I. Topological Invariant of Freestanding Bismuthene

II. Formation Mechanism of 2D Weyl Cones

III. Effective Model of 2D Weyl Semimetals in Bismuthene

IV. Spin Texture of Edge States in Bismuthene/SnSe

V. Robustness of the Topological Edge State Band

VI. STM Topography, dI/dV Mapping, and QPI Pattern

VII. Calculated Spin Texture of Weyl States in Bi/SnSe

VIII. ARPES Spectrum and Its Second Derivative of Bi/SnSe(S)

IX. Two Crystal Orientations on the Cleaved Surface of SnSe(S)

X. Extra Spin ARPES data

XI. Band Topology of Bi/SnSe under Perturbations

XII. Toy Model of Fermi String Edge States

XIII. Band Structure of Epitaxial Bismuthene in a Zeeman Field

XIV. Exotic Properties of 2D Weyl Semimetals

I. Topological Invariant of Freestanding Bismuthene

To examine the topological invariant of freestanding bismuthene, we calculated the Wannier charge center evolution.[1] The result is displayed in Supplementary Figure 1. The function $\theta(k)$ is the phase factor of eigenvalues. Therefore, the evolution of the Wannier center across the phase space can be regarded as the winding number of the Wannier center. Supplementary Figure 1 shows the topological $\mathbb{Z}_2$ invariant $\nu = 1$ as the trajectory of the Wannier center is an open curve traversing the whole Brillouin zone. The calculated Wannier center indicates that freestanding bismuthene is a 2D topological insulator.

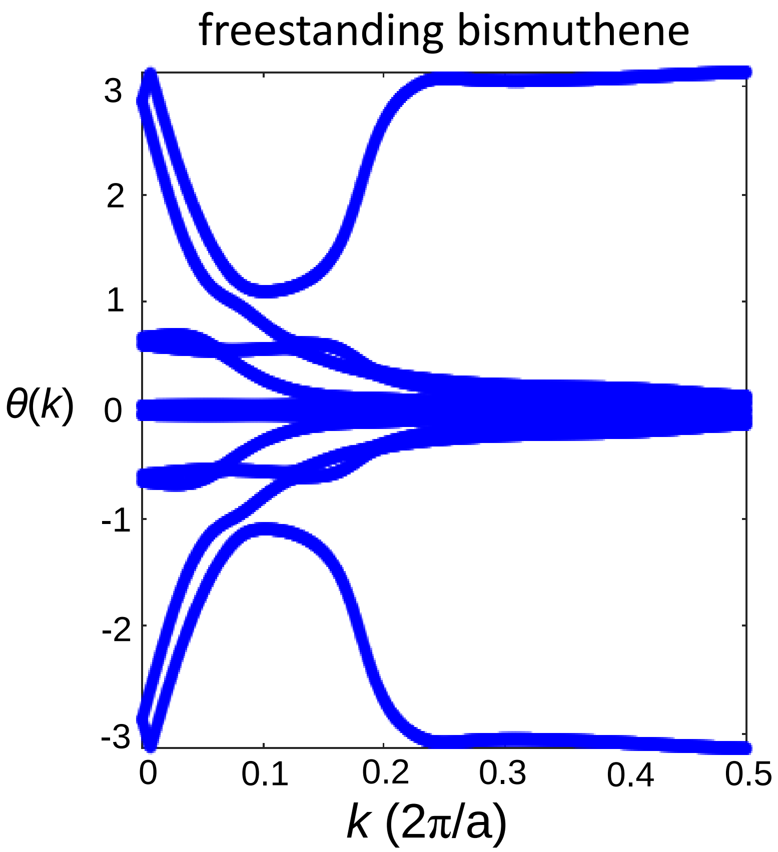

Supplementary Figure 1 : Wannier charge center evolution of freestanding bismuthene.

II. Formation Mechanism of 2D Weyl Cones

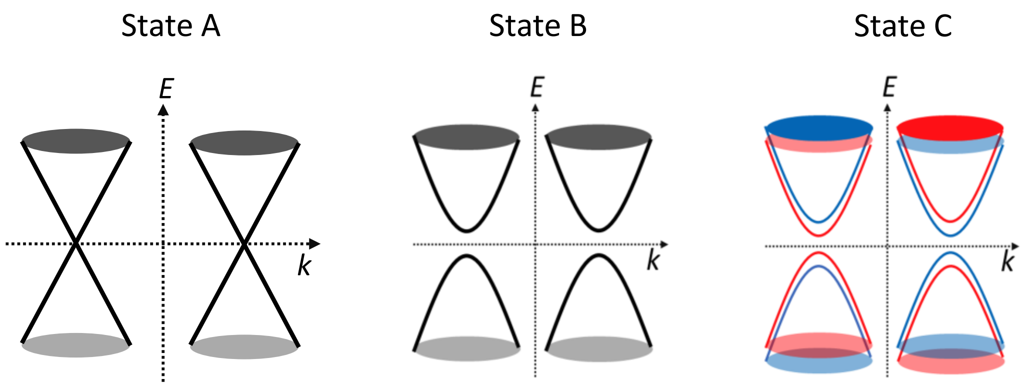

Supplementary Figure 2: Schematic of State A (gapless Dirac cones), State B (gapped Dirac cones), and State C (spin-split gapped Dirac cones). The red and blue colors in State C indicate opposite spin orientations.

a. Gapless 2D Dirac States *v.s.* Gapped 2D Dirac States

We now compare the energy of two systems: gapless 2D Dirac states and gapped 2D Dirac states, as schematically shown by State A and State B in Supplementary Figure 2. The gapless Dirac cones (State A) can be described by the effective Hamiltonian

$$H_{\mathrm{D}} = v(k_x\sigma_x + k_y\sigma_y), \tag{1}$$

where σ_i $(i = x, y)$ are the pseudospin matrices. The energy eigenvalues are $E_{\pm} = \pm vk$, where $k = \sqrt{k_x^2 + k_y^2}$.

The Hamiltonian of Dirac cones with a SOC gap (State B) can be obtained by adding a

SOC term, which is

$$H_{\mathrm{SOC}} = H_{\mathrm{D}} + \lambda_{\mathrm{SOC}}\sigma_z s_z = v(k_x\sigma_x + k_y\sigma_y) + \lambda_{\mathrm{SOC}}\sigma_z s_z, \tag{2}$$

where s_i $(i = x, y, z)$ are spin matrices and λ_{SOC} is the effective spin-orbit coupling. The energy eigenvalues are $E_{\pm}^{\mathrm{SOC}} = \pm\sqrt{v^2k^2 + \lambda_{\mathrm{SOC}}^2}$. The size of band gap is $E_{\mathrm{g}} = 2|\lambda_{\mathrm{SOC}}|$.

The total energy of occupied states can be obtained by integrating $E_{-}(k)$ over the Brillouin zone. It turns out that the energy difference between State A and State B is proportional to λ_{SOC}^2, *i.e.*

$$E_{\mathrm{SOC}}^{occu.} - E_{\mathrm{D}}^{occu.} \propto -\lambda_{\mathrm{SOC}}^2 \tag{3}$$

So the Dirac cones with SOC gaps are energetically favored, which also means that the 2D Dirac states are fragile against gap-opening effects such as SOC.

b. Gapped 2D Dirac States *v.s.* Gapped Dirac States with spin splitting

Now we introduce a spin-splitting term to the gapped Dirac fermion systems. The spin-splitting term must break the space inversion symmetry for nonmagnetic systems. One example is the dipole field from the SnSe(S) substrate. This dipole field creates a potential difference between the "A" and "B" sublattices of bismuthene. This dipole term can been effectively described by $\lambda_{\mathrm{Dip}}\sigma_z$. So the total Hamiltonian with the dipole term is

$$H_{\mathrm{SS}} = H_{\mathrm{SOC}} + \lambda_{\mathrm{Dip}}\sigma_z = v(k_x\sigma_x + k_y\sigma_y) + \lambda_{\mathrm{SOC}}\sigma_z s_z + \lambda_{\mathrm{Dip}}\sigma_z, \tag{4}$$

where “SS" means spin-splitting because the substrate dipole term breaks the spin degeneracy of the Dirac bands as shown by State C in Supplementary Figure 2. The energy eigenvalues

are

$$E^1_{\pm} = \pm\sqrt{v^2k^2 + (\lambda_{\text{SOC}} - \lambda_{\text{Dip}})^2} \tag{5}$$

$$E^2_{\pm} = \pm\sqrt{v^2k^2 + (\lambda_{\text{SOC}} + \lambda_{\text{Dip}})^2}. \tag{6}$$

We have two sets of gapped Dirac cones: one with a reduced gap of $2|\lambda_{\text{SOC}} - \lambda_{\text{Dip}}|$ and the other with an enlarged gap of $2|\lambda_{\text{SOC}}+\lambda_{\text{Dip}}|$ (suppose λ_{SOC} and λ_{Dip} have the same sign). The spin of the two Dirac cones is polarized in opposite directions as shown in Supplementary Figure 2. The energy gap between the bands described by $E^1_{\pm}$ vanishes when $\lambda_{\text{SOC}} = \lambda_{\text{Dip}}$.

Now let us compare the energy of the gapped Dirac fermion systems without (State B) and with (State C) the substrate perturbation. A straightforward calculation shows that

$$E^{occu.}_{\text{SS}} - E^{occu.}_{\text{SOC}} \propto -\lambda^2_{\text{Dip}} \tag{7}$$

So the Dirac bands with spin splitting are energetically favored. This is because the energy reduction by forming the $E^2_{\pm}$ bands is greater than the energy cost for forming the $E^1_{\pm}$ bands. The result suggests that the system tends to relax towards a configuration with larger values of λ_{Dip}. The argument also works for other substrate couplings such as λ_{Dip} as discussed in the main text.

c. Spontaneous relaxation towards a 2D Weyl semimetal

Consider a bismuthene layer floating on the surface of SnSe(S), the Bi layer relaxes to narrow down the SOC gap and consequently lower the electronic energy of the system. The SnSe(S) substrate surface provides a sufficiently strong substrate perturbation to minimize the band gap of bismuthene, leading to a gapless 2D Weyl semimetal state. This spontaneous process of gap minimization can also be seen in the first-principles band structure shown in Supplementary Figure 3. The freestanding bismuthene possesses a band gap of 0.1 eV

(Supplementary Figure 3**a**). The band gap is reduced to 20 meV due to the Rashba splitting in the partially relaxed heterostructure of bismuthene and SnS (Supplementary Figure 3**b**). In this heterostructure, only the interlayer spacing between bismuthene and SnS is optimized while the lattice of bismuthene is fixed to be the freestanding one. The remaining small band gap is eliminated in a fully relaxed bismuthene lattice on the SnS surface as shown in Supplementary Figure 3**c**. The gapless Weyl cones are also found in the band structure of a fully relaxed Bi/SnSe heterostructure (Supplementary Figure 3**d**).

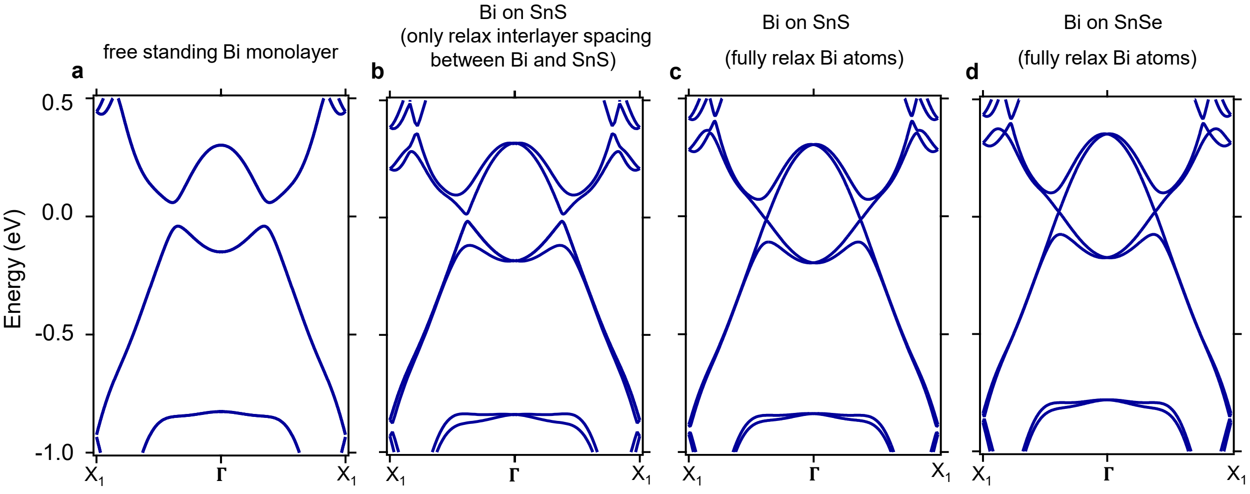

Supplementary Figure 3: Calculated band structure along $X_1-\Gamma-X_1$ direction of **a** freestanding bismuthene, **b** bismuthene on SnS with only interlayer spacing relaxed, **c** bismuthene on SnS with Bi atoms fully relaxed, and **d** bismuthene on SnSe with Bi atoms fully relaxed.

III. Effective Model for 2D Weyl Semimetals in Bismuthene

The lattice structure of bismuthene possesses the symmetry of the $Pmna$ space group (No. 53). The generators of the $Pmna$ group include a twofold rotation $C_{2z} \equiv \{C_{2z}|\frac{1}{2}\frac{1}{2}\}$, a glide line $G_y \equiv \{M_y|\frac{1}{2}\frac{1}{2}\}$, and the inversion $\mathcal{P}$ symmetries. We also have a vertical mirror $M_x = C_{2z}G_y$. Since α-bismuthene is nonmagnetic, the time-reversal symmetry $\mathcal{T}$ is also included.

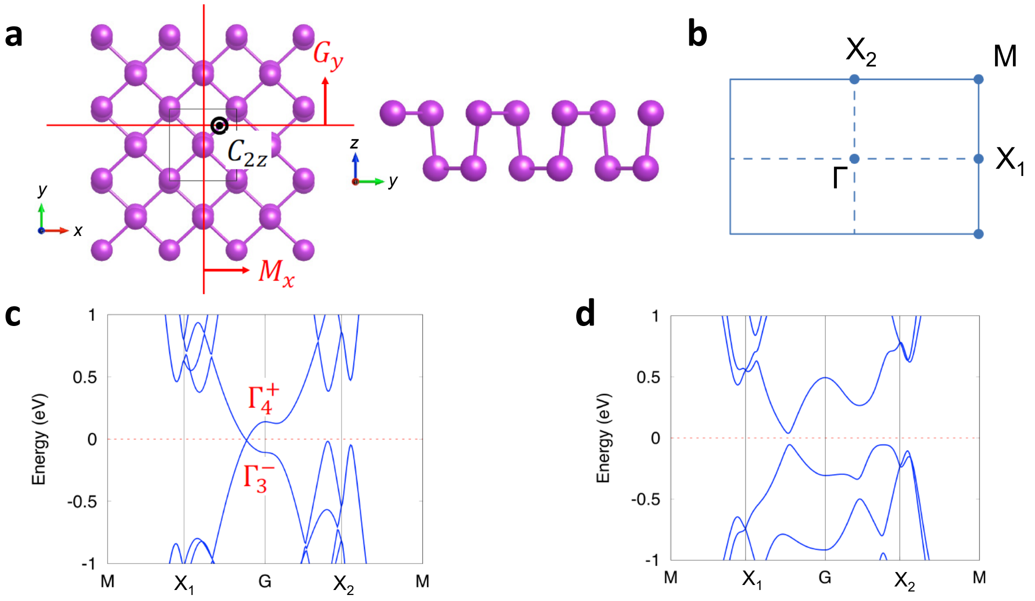

Supplementary Figure 4: **a** Crystal structure of bismuthene. **b** Brillouin zone of bismuthene. **c** Band structure in the absence of SOC. **d** Band structure in the presence of SOC.

Dirac points without spin-orbit coupling. — First, we investigate the band structure of bismuthene in the absence of spin-orbit coupling (SOC), as shown in Supplementary Figure 4**c**. A pair of Dirac points appears along Γ-X_1 path, which is formed by the band inversion at Γ. To capture the feature of Dirac points, we can construct the effective model of Dirac points around Γ.

The little co-group at Γ is the D_{2h} point group, which includes the generators: a twofold rotation C_{2z}, a mirror M_y, and the inversion $\mathcal{P}$. Note that M_y is just the rotation part

Supplementary Table 1: Character table for the single-valued point group D_{2h}

	E	C_{2z}	C_{2y}	C_{2x}	$\mathcal{P}$	M_z	M_y	M_x	functions
Γ_1^+	1	1	1	1	1	1	1	1	-
Γ_2^+	1	-1	1	-1	1	-1	1	-1	xz
Γ_3^+	1	1	-1	-1	1	1	-1	-1	xy
Γ_4^+	1	-1	-1	1	1	-1	-1	1	yz
Γ_1^-	1	1	1	1	-1	-1	-1	-1	-
Γ_2^-	1	-1	1	-1	-1	1	-1	1	y
Γ_3^-	1	1	-1	-1	-1	-1	1	1	z
Γ_4^-	1	-1	-1	1	-1	1	1	-1	x

of G_y. We also need to consider $\mathcal{T}$. The crossing bands correpond to the Γ_4^+ and Γ_3^- irreducible representations of D_{2h}. Since the low-energy bands are dominated with Bi-p_z orbitals, we may consider the p_z orbital on two sublattices A and B, which we denote them as $\{|A, p_z\rangle, |B, p_z\rangle\}$. Assuming that C_{2z}, M_y, and $\mathcal{P}$ all map the sublattice A to B, the combinatorial states $\{\frac{\sqrt{2}}{2}|A, p_z\rangle - \frac{\sqrt{2}}{2}|B, p_z\rangle, \frac{\sqrt{2}}{2}|A, p_z\rangle + \frac{\sqrt{2}}{2}|B, p_z\rangle\}$ correspond to Γ_4^+ and Γ_3^-, respectively. Hence, we can use the states $\{|A, p_z\rangle, |B, p_z\rangle\}$ as a basis to construct the effective Hamiltonian around Γ.

Under the basis, the generators and $\mathcal{T}$ can be represented as

$$C_{2z} = \sigma_x, \qquad M_y = \sigma_x, \qquad \mathcal{P} = -\sigma_x, \qquad \mathcal{T} = \mathcal{K} \tag{8}$$

Constrained by these symmetries, the effective Hamiltonian around Γ can be obtained as (up to the second order),

$$\mathcal{H}_0^\Gamma(\boldsymbol{k}) = \epsilon(\boldsymbol{k})\sigma_0 + c_1 k_y \sigma_y + (c_2 k_x^2 + c_3 k_y^2)\sigma_x + \Delta\sigma_x \tag{9}$$

where $\epsilon(\boldsymbol{k}) = \epsilon_0 + \epsilon_1 k_x^2 + \epsilon_2 k_y^2$, σ_i are Pauli matrices, and ϵ_i, c_i, Δ are real parameters. The band inversion occurs when $\Delta < 0$ and $k_y = 0$, which gives rise to a pair of Dirac points at $(\pm\sqrt{-\Delta/c_2}, 0)$ along the k_x axis.

Expanding around each Dirac point, we can obtain the low-energy Hamiltonian for Dirac

points, which reads

$$\mathcal{H}_0^{\mathrm{Dirac}}(\boldsymbol{q}) = \omega(\boldsymbol{q})\sigma_0 + \tau v_x q_x \sigma_x + v_y q_y \sigma_y \tag{10}$$

where $\omega(\boldsymbol{q}) = \omega_0 + \tau\omega_1 q_x$ with $\omega_0 = \epsilon_0 - \epsilon_1\Delta/c_2$, and $\omega_1 = 2\epsilon_1\sqrt{-\Delta/c_2}$, which gives rise to a tilting of the Dirac cone in the q_x direction. Here, $v_x = 2\sqrt{-\Delta c_2}$, $v_y = c_1$, $\tau = \pm 1$ represents the chirality of each Dirac point.

Spinful case. — When SOC is included, the generators of D_{2h} and $\mathcal{T}$ at Γ can be expressed as

$$C_{2z} = -i\sigma_x s_z, \qquad M_y = -i\sigma_x s_y, \qquad \mathcal{P} = -\sigma_x, \qquad \mathcal{T} = -is_y\mathcal{K} \tag{11}$$

which constrain the effective Hamiltonian to

$$\mathcal{H}_{\mathrm{SOC}}^{\Gamma}(\boldsymbol{k}) = \mathcal{H}_0^{\Gamma}(\boldsymbol{k}) + c_4 k_x \sigma_z s_z \tag{12}$$

Apparently, the Dirac point is violated by the SOC term $c_4 k_x \sigma_z s_z$. Expanding around $(\pm\sqrt{-\Delta/c_2}, 0)$, the low-energy Hamiltonian can be obtained as (omitting the k-dependent SOC term)

$$\mathcal{H}_{\mathrm{SOC}}^{\mathrm{Dirac}}(\boldsymbol{q}) = \mathcal{H}_0^{\mathrm{Dirac}}(\boldsymbol{q}) + \tau\lambda_{\mathrm{SOC}}\sigma_z s_z \tag{13}$$

where $\lambda_{\mathrm{SOC}} = c_4\sqrt{-\Delta/c_2}$. From the effective Hamiltonian, the band gap at $(\pm\sqrt{-\Delta/c_2}, 0)$ is $2|\lambda_{\mathrm{SOC}}|$.

Epitaxial bismuthene on SnS(Se). — Next, we place bismuthene on the SnS(Se) substrate. The substrate has two types of effects on the Hamiltonian of α-bismuthene: the sublattice bias potential due to the in-plane dipolar field of SnS(Se), and the Rashba term due to the vertical electric field on SnS(Se) surface. The former preserves the horizontal glide mirror $G_z = C_{2z}\mathcal{P}$ but introduces a new term to the Hamiltonian

$$H_{\mathrm{Dip}}(\boldsymbol{k}) = \lambda_{\mathrm{Dip}}\sigma_z, \tag{14}$$

while the vertical electric field breaks G_z. The Rashba term constrained by symmetry is given by (up to the first order)

$$H_{\mathrm{R}}(\boldsymbol{k}) = c_5\sigma_y s_x + (c_6 k_x s_y + c_7 k_y s_x) + (c_8 k_x \sigma_x s_y + c_9 k_y \sigma_x s_x) \tag{15}$$

Hence, the effective Hamiltonian is given as

$$\begin{aligned}\mathcal{H}^{\Gamma}(\boldsymbol{k}) &= \mathcal{H}^{\Gamma}_{\mathrm{SOC}}(\boldsymbol{k}) + H_{\mathrm{Dip}}(\boldsymbol{k}) + H_{\mathrm{R}}(\boldsymbol{k}) \\ &= \epsilon(\boldsymbol{k})\sigma_0 + c_1 k_y \sigma_y + (c_2 k_x^2 + c_3 k_y^2)\sigma_x + \Delta\sigma_x + c_4 k_x \sigma_z s_z + \lambda_{\mathrm{Dip}}\sigma_z \\ &\quad + c_5\sigma_y s_x + (c_6 k_x s_y + c_7 k_y s_x) + (c_8 k_x \sigma_x s_y + c_9 k_y \sigma_x s_x)\end{aligned} \tag{16}$$

Expanding around $(\pm\sqrt{-\Delta/c_2}, 0)$, the low-energy Hamiltonian can be obtained as (omitting the k-dependent Rashba term)

$$\mathcal{H}^{\mathrm{Dirac}}(\boldsymbol{q}) = \omega(\boldsymbol{q})\sigma_0 + (\tau v_x q_x \sigma_x + v_y q_y \sigma_y) + \tau\lambda_{\mathrm{SOC}}\sigma_z s_z + \lambda_{\mathrm{Dip}}\sigma_z + \lambda_{\mathrm{V}}\sigma_y s_x + \tau\lambda'_{\mathrm{V}}\sigma_x s_y \tag{17}$$

with $\lambda_{\mathrm{V}} = c_5$ and $\lambda'_{\mathrm{V}} = c_8\sqrt{-\Delta/c_2}$. The two Rashba couplings λ_{V} and λ'_{V} are not equal in general because they are not related to each other by crystal symmetries.

Now, we examine the eigenvalues at $(q_x, q_y) = (0, 0)$. Here are several typical cases:

- If the Rashba terms are zero ($\lambda_{\mathrm{V}} = 0, \lambda'_{\mathrm{V}} = 0$), the eigenvalues can be given as

$$\varepsilon_1 = \omega_0 + \lambda_{\mathrm{SOC}} + \lambda_{\mathrm{Dip}} \tag{18}$$

$$\varepsilon_2 = \omega_0 + \lambda_{\mathrm{SOC}} - \lambda_{\mathrm{Dip}} \tag{19}$$

$$\varepsilon_3 = \omega_0 - \lambda_{\mathrm{SOC}} + \lambda_{\mathrm{Dip}} \tag{20}$$

$$\varepsilon_4 = \omega_0 - \lambda_{\mathrm{SOC}} - \lambda_{\mathrm{Dip}} \tag{21}$$

  and apparently, the gap closes when $|\lambda_{\mathrm{SOC}}| = |\lambda_{\mathrm{Dip}}|$.

- If one of the Rashba terms is zero, say $\lambda'_{\rm V} = 0$, the eigenvalues can be given as

$$\varepsilon_1 = \omega_0 + \lambda_{\rm SOC} + \sqrt{\lambda_{\rm Dip}^2 + \lambda_{\rm V}^2} \tag{22}$$

$$\varepsilon_2 = \omega_0 + \lambda_{\rm SOC} - \sqrt{\lambda_{\rm Dip}^2 + \lambda_{\rm V}'^2} \tag{23}$$

$$\varepsilon_3 = \omega_0 - \lambda_{\rm SOC} + \sqrt{\lambda_{\rm Dip}^2 + \lambda_{\rm V}^2} \tag{24}$$

$$\varepsilon_4 = \omega_0 - \lambda_{\rm SOC} - \sqrt{\lambda_{\rm Dip}^2 + \lambda_{\rm V}^2} \tag{25}$$

  and the gap closes at $(0, 0)$ when $|\lambda_{\rm SOC}| = \sqrt{\lambda_{\rm Dip}^2 + \lambda_{\rm V}'^2}$.

- Generally, the eigenvalues can be given as

$$\varepsilon_1 = \omega_0 + \lambda_{\rm SOC} + \sqrt{\lambda_{\rm Dip}^2 + (\lambda_{\rm V} + \lambda'_{\rm V})^2} \tag{26}$$

$$\varepsilon_2 = \omega_0 + \lambda_{\rm SOC} - \sqrt{\lambda_{\rm Dip}^2 + (\lambda_{\rm V} + \lambda'_{\rm V})^2} \tag{27}$$

$$\varepsilon_3 = \omega_0 - \lambda_{\rm SOC} + \sqrt{\lambda_{\rm Dip}^2 + (\lambda_{\rm V} - \lambda'_{\rm V})^2} \tag{28}$$

$$\varepsilon_4 = \omega_0 - \lambda_{\rm SOC} - \sqrt{\lambda_{\rm Dip}^2 + (\lambda_{\rm V} - \lambda'_{\rm V})^2} \tag{29}$$

  and the gap closes when $|\lambda_{\rm SOC}| = \frac{1}{2}\left(\sqrt{\lambda_{\rm Dip}^2 + (\lambda_{\rm V} + \lambda'_{\rm V})^2} + \sqrt{\lambda_{\rm Dip}^2 + (\lambda_{\rm V} - \lambda'_{\rm V})^2}\right)$.

The features induced by the sublattice dipolar term and the Rashba terms can reflected on the spin texture of the band structure. In the case that the Rashba terms are set to be zero, the direction of spin is constrained along $\hat{z}$ direction, since $[G_z, \sigma_z] = 0$. When the Rashba term is further turned on, the components of spin within the x-y plane become nonzero. Specifically, for nonzero $\lambda_{\rm V}$ and $\lambda'_{\rm V}$, the y component of spin is nonzero along Γ-X_1 path, while the x component of spin is nonzero along k_y direction passing through the Weyl point.

IV. Spin Texture of Edge States in Bismuthene/SnSe

The edge state bands including the 1D topological edge band are spin polarized, which is shown in the calculated spin texture of edge state bands in Supplementary Figure 5. The

spin components $\langle s_y \rangle$ and $\langle s_z \rangle$ change the sign as the edge state band disperses, because the spin is not a good quantum number in the presence of SOC.

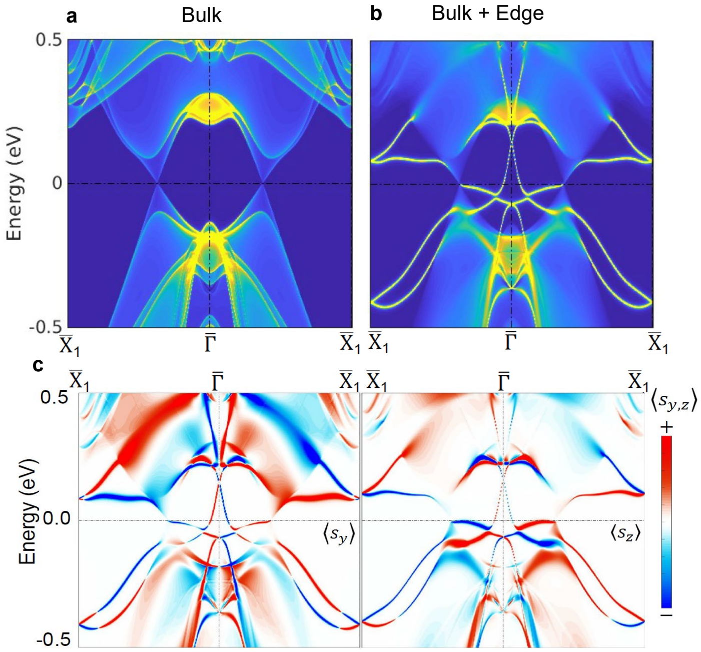

Supplementary Figure 5: **a,** Projected bulk bands of bismuthene/SeSe. **b,** Band spectrum of a semi-infinite bismuthene/SeSe with an open edge along the (010) direction. **c,** Spin components $\langle s_y \rangle$ and $\langle s_z \rangle$ of the bulk and edge bands shown in Fig. 5**a** of the main text. The $\langle s_x \rangle$ component vanishes because $\overline{\Gamma}$-$\overline{\mathrm{X}}_1$ lies in a mirror plane.

V. Robustness of the Topological Edge State Band

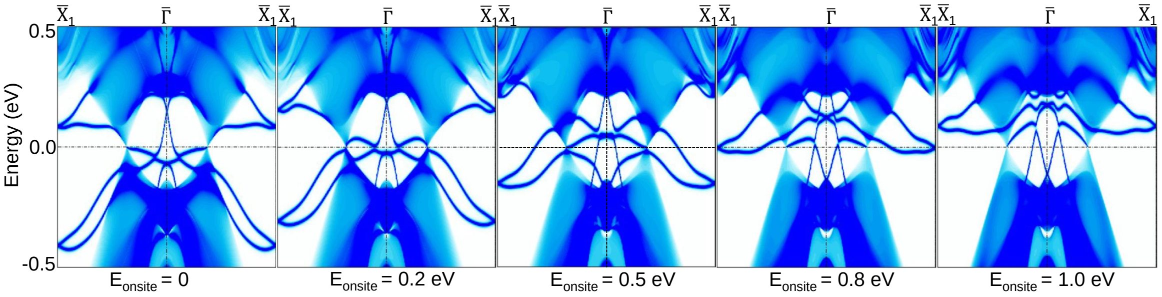

Supplementary Figure 6: The edge bands and bulk bands of a semi-infinite bismuthene/SnSe heterostructure with an open boundary in the (010) direction. The bands are weighted with the charge density near the edge. An onsite potential energy E_{onsite} (0, 0.2, 0.5, 0.8, and 1.0 eV) is added to the two outermost Bi sites on the edge.

To see the robustness of the topological edge state band, we simulate the band spectrum with altered boundary conditions. Specifically, an onsite potential energy E_{onsite} is added to the two outermost Bi sites on the edge. The calculated edge band spectrum is shown in Supplementary Figure 6. Though the dispersion of edge bands is sensitive to the boundary condition, one edge state band is always connected to the two bulk Weyl nodes. The result reflects the fact that the connection of the topological edge state band to Weyl nodes is topologically protected.

VI. STM Topography, dI/dV Mapping, and QPI Pattern of Bi/SnSe(S)

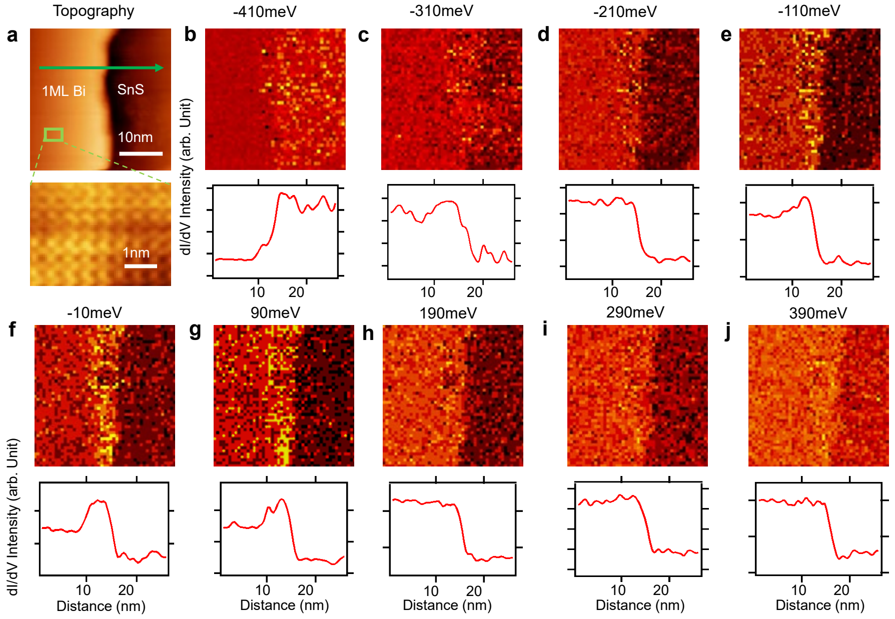

Supplementary Figure 7: **a,** STM topography of bismuthene grown on SnS taken at room temperature. **b-j,** dI/dV mapping (top) over the area shown in **a** and the dI/dV spectrum (averaged in the direction parallel to the edge) under bias voltage from -410 meV to 390 meV. A pronounced local density of states (LDOS) is seen near the edge around zero bias voltage.

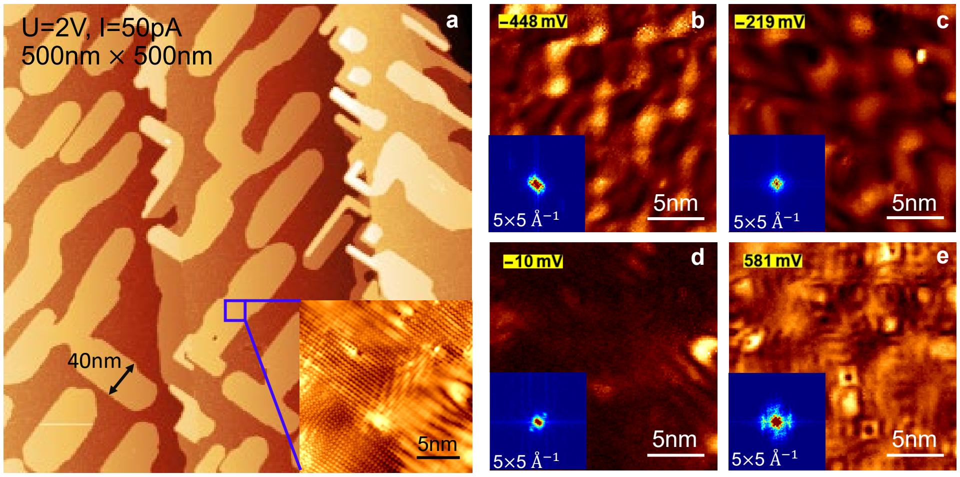

Supplementary Figure 8: **a,** STM topography of bismuthene grown on SnSe. **b-e,** dI/dV mapping and the quasiparticle interference (QPI) pattern (shown in the insets) under different bias voltages.

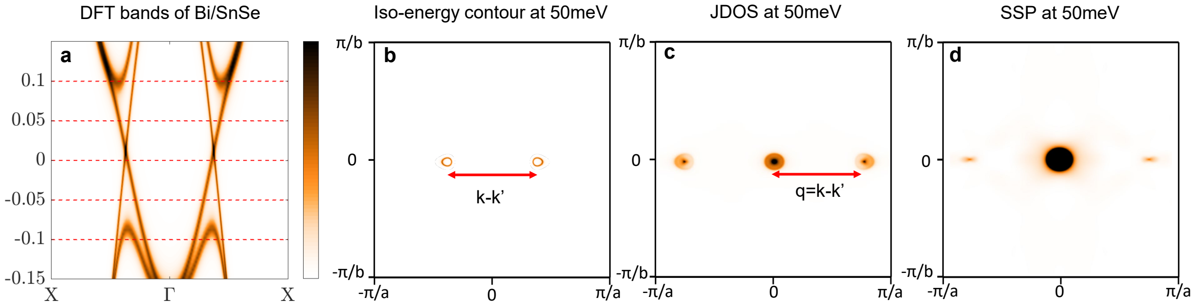

Supplementary Figure 9: **a,** Calculated band structure of Bi/SnSe. **b,** Calculated iso-energy contour at $E = 50$ meV. **c,** Joint density of states (JDOS) at $E = 50$ meV. **d,** The spin-dependent scattering probability (SSP) at $E = 50$ meV.

VII. Calculated Spin Texture of Weyl States in Bi/SnSe

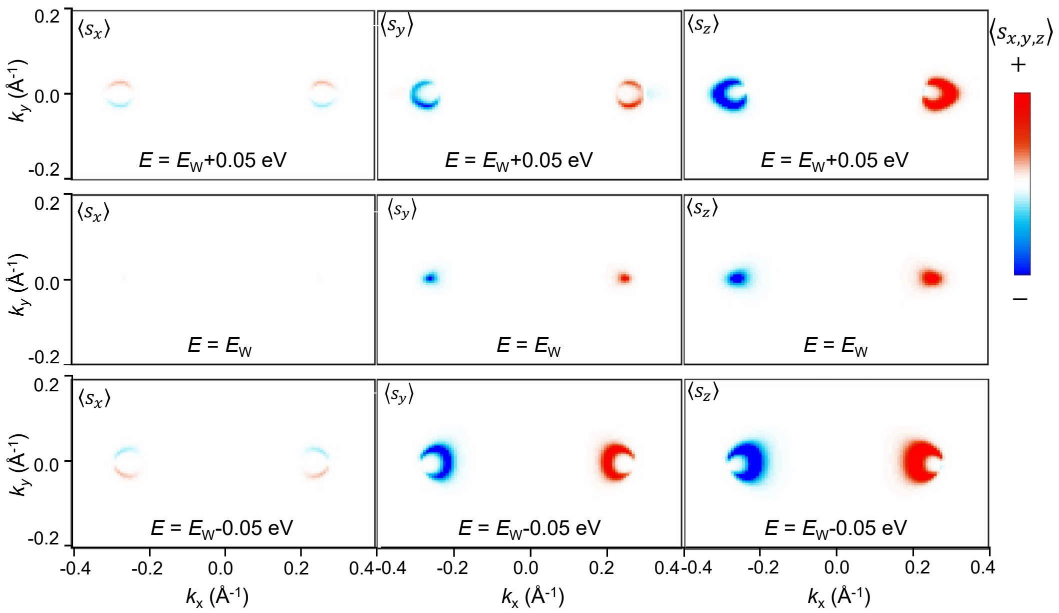

Supplementary Figure 10: Calculated spin texture of iso-energy contours at $E = E_\mathrm{W}$ - 0.05 eV, E_W, and E_W + 0.05 eV, where E_W is the energy of the Weyl nodal points. The calculated spectral intensity is plotted with the inclusion of photoemission matrix elements.

VIII. ARPES Spectrum and Its Second Derivative of Bi/SnSe and Bi/SnS

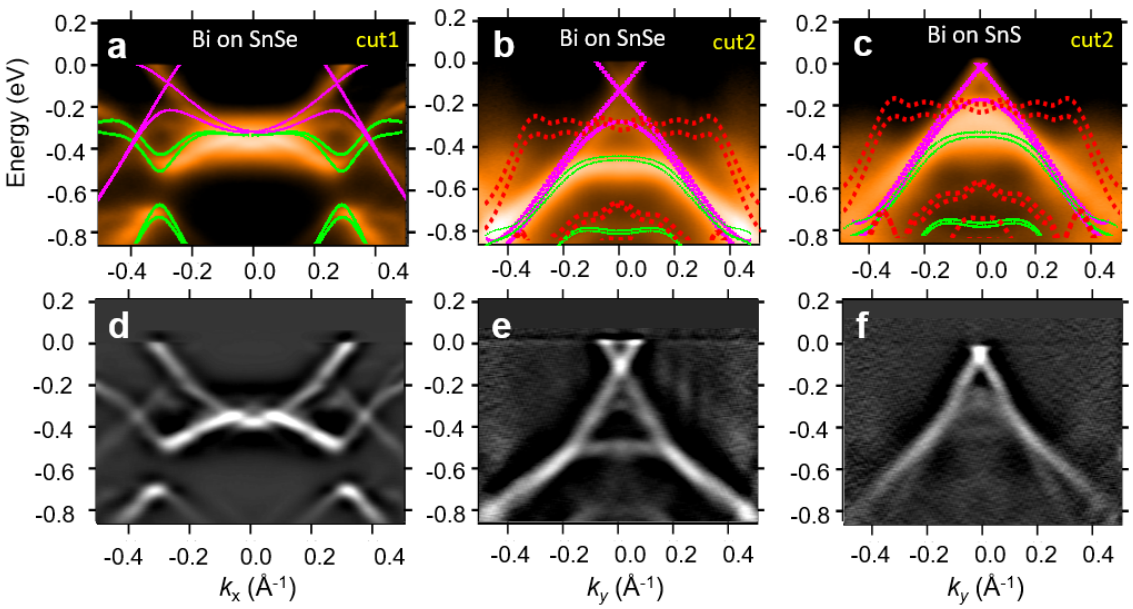

Supplementary Figure 11: **a,** ARPES spectrum of Bi/SnSe along "cut1". The magenta lines are calculated bands in the direction of Γ-X_1 while the green lines are bands along Γ-X_2. **b,** ARPES spectrum of Bi/SnSe along "cut2". The magenta lines are calculated bands along "cut2" while the green lines are bands in the direction perpendicular to "cut2". The dotted lines depict the calculated band structure of 2ML bismuthene on SnSe. **c,** same as **b** but for Bi/SnS. **d-f,** The second derivative of ARPES spectra shown in **a-c**.

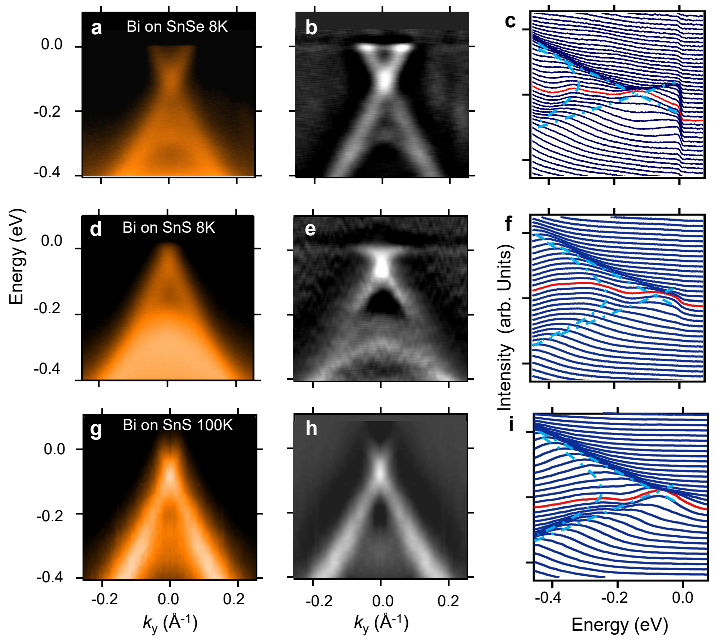

Supplementary Figure 12: **a,** ARPES spectrum of Bi/SnSe along "cut2" taken at T=8K. **b,** The second derivative of ARPES spectra shown in **a**. **c,** The energy distribution curve (EDC)-analysis of ARPES spectra shown in **a**. **d-f,** Same as **a-c**, but for Bi/SnS taken at T=8K. **g-i,** Same as **a-c**, but for Bi/SnS taken at T=100K.

IX. Two Orientations of Bismuthene on the Cleaved Surface of SnSe(S)

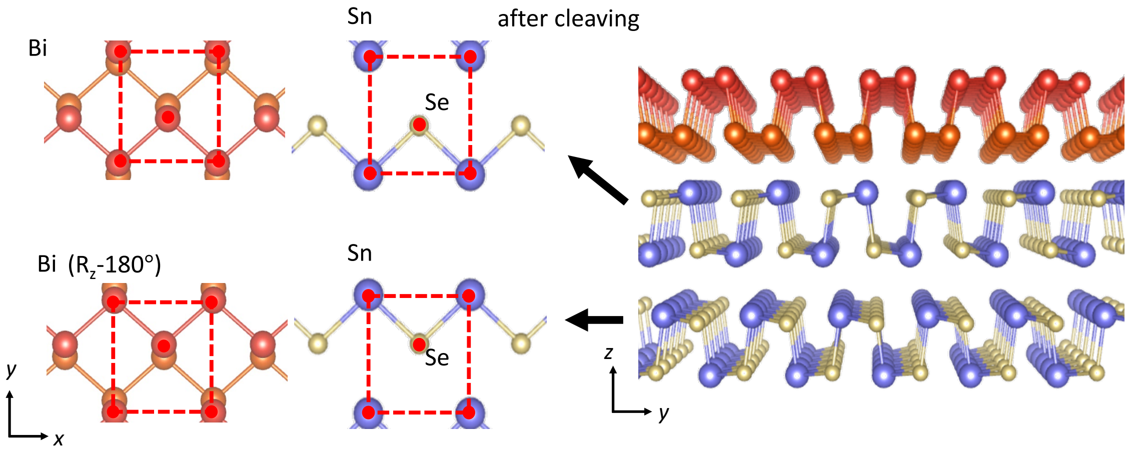

Supplementary Figure 13: Schematic of the two possible orientations of bismuthene films grown on the cleaved surface of SnSe(S)

There are two possible surface unit cells of SnSe from the top layer and the second layer from the top, as marked by the black arrows in Supplementary Figure 13. Given the weak vdW interlayer coupling of SnSe, the two surface unit cells are likely to appear after cleaving. The bismuthene monolayer films grown on the two configurations of substrate surface are related by a 180° rotation about the z axis. The rotation changes the sign of spin polarization $\langle s_z \rangle$ of the Weyl states while has no effects on the other two components $\langle s_{x,y} \rangle$ of spin. This can be seen in the following equations,

$$\langle s_z \rangle_{\mathrm{R}}(\boldsymbol{k}) = \langle \psi(-\boldsymbol{k}) | \hat{s}'_z | \psi(-\boldsymbol{k}) \rangle = \langle \psi(-\boldsymbol{k}) | \hat{s}_z | \psi(-\boldsymbol{k}) \rangle = \langle s_z \rangle(-\boldsymbol{k}) = -\langle s_z \rangle(\boldsymbol{k}), \quad (30)$$

$$\langle s_{x,y} \rangle_{\mathrm{R}}(\boldsymbol{k}) = \langle \psi(-\boldsymbol{k}) | \hat{s}'_{x,y} | \psi(-\boldsymbol{k}) \rangle = \langle \psi(-\boldsymbol{k}) | -\hat{s}_{x,y} | \psi(-\boldsymbol{k}) \rangle = -\langle s_{x,y} \rangle(-\boldsymbol{k}) = \langle s_{x,y} \rangle(\boldsymbol{k}), \quad (31)$$

where $\langle s_i \rangle_{\mathrm{R}}$, $(i = x, y, z)$ are the spin polarization components of the rotated bismuthene film and $\hat{s}'_i$, $(i = x, y, z)$ are spin operators under the rotation operation.

X. Extra Spin ARPES data

a. Canted spin texture: theory and experiment

Here we present the spin-ARPES data taken from a different Bi/SnSe sample. The spin result is consistent with that shown in Fig. 3 of the main text. The calculated spin texture is plotted in Supplementary Figure 14**g**. The magnitude of the observed spin polarization $\langle s_z \rangle$ is suppressed due to the existence of rotated domains in the MBE samples (see the previous section). Nonetheless, the observed $\langle s_z \rangle$ exhibits a similar characteristic to the $\langle s_y \rangle$ component, consistent with the first-principles results. The spin-ARPES results demonstrate the canted spin texture of Weyl fermion states.

b. Spin Polarization above/below the Weyl Node and Experimental Geometry

c. Error analysis of spin polarization data

The spin polarization results are extracted from electron signals collected by the "+" and "−" channels of the spin detector at each angle for a fixed period of time. We assumed the number of the collected electrons follows the Poisson statistics, so the standard deviation $\sigma_I = \sqrt{I}$, where I is the intensity of the electron signals in the spin channels. Using the definition of spin polarization P and the standard deviation σ_P, we can get the relative statistical errors in the spin results:

$$\sigma_P = \frac{1}{S_{\text{eff}}}\sqrt{\left(\frac{\sigma_{I_+ - I_-}}{I_+ - I_-}\right)^2 - \left(\frac{\sigma_{I_+ + I_-}}{I_+ + I_-}\right)^2} = \frac{1}{S_{\text{eff}}}\frac{1}{\sqrt{|I_+ - I_-|}} \tag{32}$$

where S_{eff}=0.275 is the effective Sherman function of our spin detector.

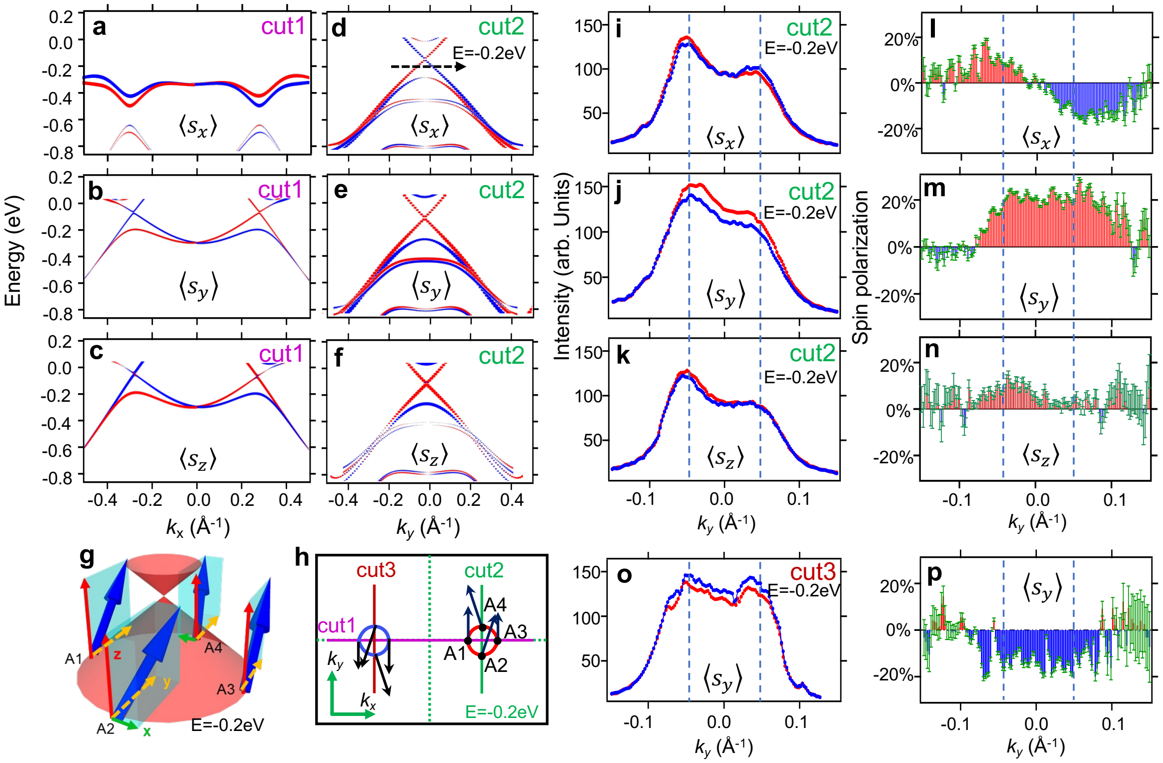

Supplementary Figure 14: **Spin texture of 2D Weyl cones in bismuthene on SnSe.a-c** The calculated spin components $\langle s_x \rangle$, $\langle s_y \rangle$, and $\langle s_z \rangle$ of the bismuthene bands along "cut1" marked in **h**, respectively. The blue and red colors represent the "spin-down" and "spin-up" states of the corresponding spin component. **d-f** Same as **a-c**, but for the line of "cut2" marked in **h**. **g** Calculated spin orientation of states A1-A4 marked in **h**. The length of the arrow indicates the magnitude of spin polarization. **h** In-plane spin texture of iso-energy contours at $E = -0.2$ eV. **i-k** Spin-resolved momentum distribution curves (MDC) taken at $E = -0.2$ eV along the line marked by the black dashed arrow in **d**. The blue and red curves are photoemission intensity recorded in the "spin-down" and "spin-up" channels, respectively, for $\langle s_x \rangle$, $\langle s_y \rangle$, and $\langle s_z \rangle$. **l-n** The shaded area with error bars indicates net spin polarization of $\langle s_x \rangle$, $\langle s_y \rangle$, and $\langle s_z \rangle$, respectively, which is calculated based on the spin results in **i-k**. **o,p** Spin-resolved MDC and calculated spin polarization of $\langle s_y \rangle$ at $E = -0.2$ eV along "cut3" from the other valley.

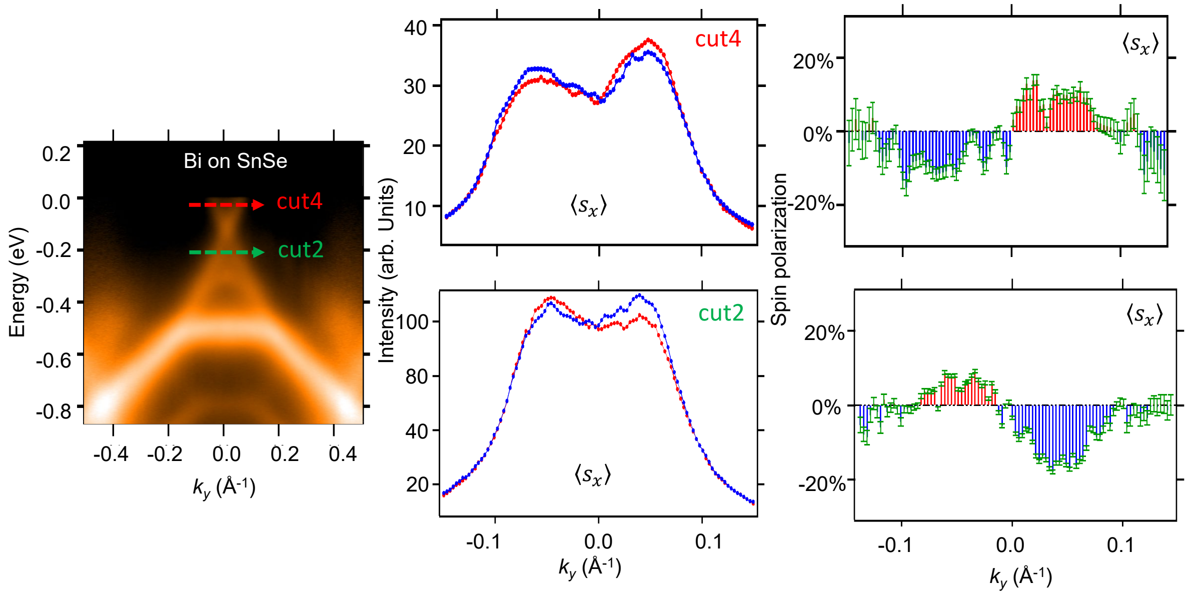

Supplementary Figure 15: Left: ARPES spectrum of the Weyl cone in Bi/SnSe (left). Middle: Spin-resolved momentum distribution curves (MDC) taken along "cut2" below the Weyl point and "cut4" above the Weyl point. The blue and red curves are photoemission intensity recorded in the "spin-down" and "spin-up" channels for $\langle s_x \rangle$. Right: The shaded area with error bars indicate the measured net spin polarization of $\langle s_x \rangle$. The result is consistent with the calculated spin polarization shown in Fig. 3 of the main text.

d. Experimental geometry of spin-resolved ARPES

LHP-polarized laser light was employed in all the ARPES and spin-ARPES measurements. We didn't notice any difference in the band shape with LVP, LCP, and RCP polarization modes. (LHP, LVP, LCP, and RCP stand for linear horizontal polarization, linear vertical polarization, left-handed circular polarization, and right-handed circular polarization, respectively.) The spectral intensity distribution is slightly different with different light polarization modes. We only showed the LHP-polarized data in this work. The experimental geometry of spin-resolved ARPES is shown in the following figure.

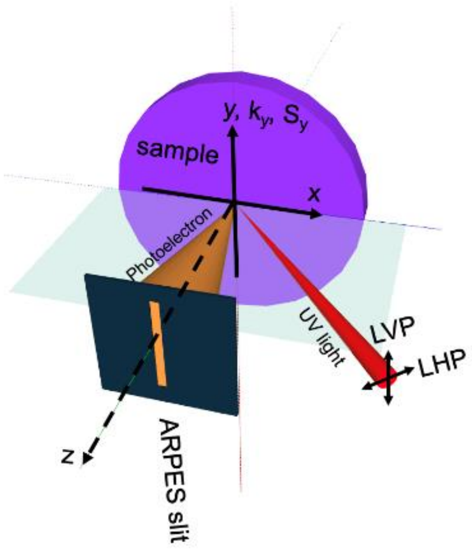

Supplementary Figure 16: The experimental geometry of spin-resolved ARPES

XI. Band Topology of Bi/SnSe under Perturbations

The 2D Weyl semimetal is at a critical point in connection to two topologically distinct insulator phases, which can be discerned in the configuration of the edge state bands (ESB), see Supplementary Figure 17. The ESBs form degenerate Kramers pairs at $\overline{\Gamma}$ as required by the time-reversal symmetry. In the 2D Weyl semimetal (Supplementary Figure 17**b**), there exist four ESBs around $\overline{\Gamma}$. The Fermi string ESBs connect to the Weyl nodes. The existence of the extra ESBs is related to the $\mathbb{Z}_2$ topological invariant when a bulk band gap is introduced to epitaxial bismuthene. The schematic of ESB dispersion is plotted in Supplementary Figure 17**e**. The ESBs hybridize with each other and open a tiny energy gap at the crossing points highlighted by the magenta dashed circles, because the band crossing is located at generic momentum and thus not protected by the time-reversal symmetry. If we increase the substrate perturbation by reducing the distance between bismuthene and the substrate surface, an energy gap is opened at the Weyl nodes, as shown in Supplementary Figure 17**c**. The inset shows that the "Fermi string" ESB stems from the bulk conduction band. As a result, the Fermi string bands together with other ESBs form two pairs of

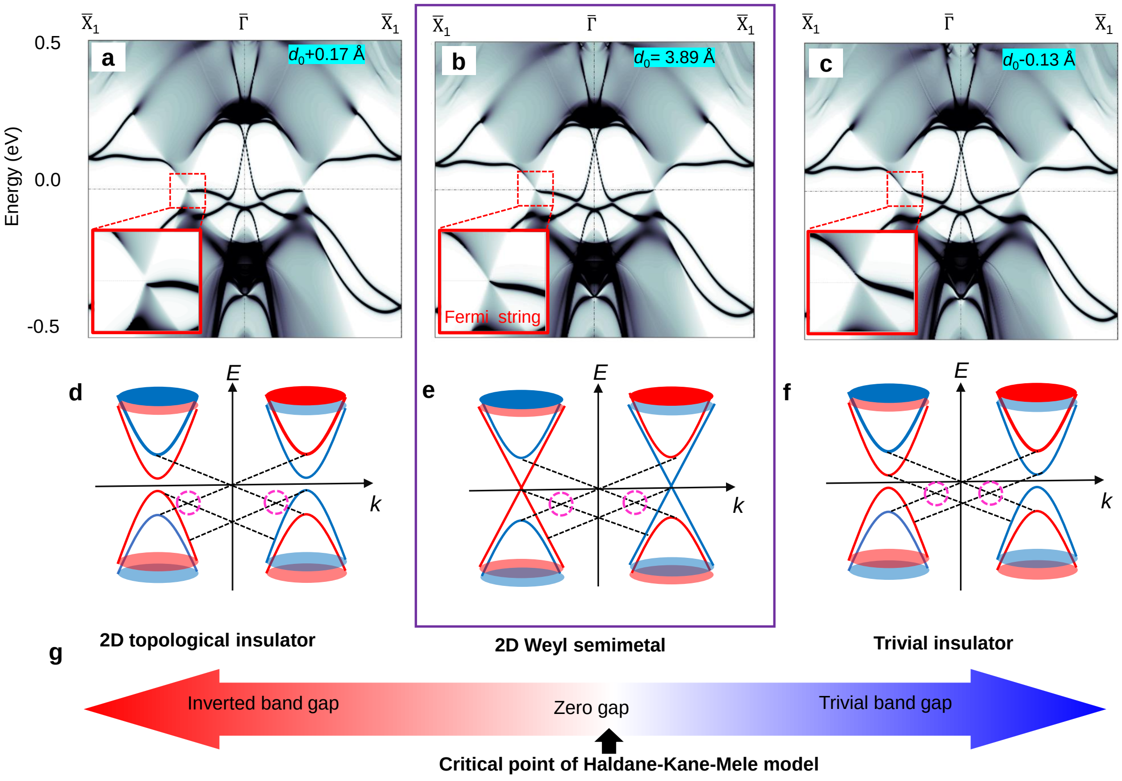

Supplementary Figure 17: **a-c,** The edge state bands and bulk bands of a semi-infinite bismuthene film on SnSe with an open boundary in the (010) direction. The bands are weighted with the charge density near the edge. The distance between bismuthene and the surface of SnSe is $d_0 + 0.17$Å, d_0, and $d_0 - 0.13$Å for **a-c**, respectively, where the equilibrium interlayer distance $d_0 = 3.89$Å. **d-f,** Schematics of connection pattern between the edge state bands and the bulk bands shown in **a-c**. The edge state bands are plotted with the dashed lines. **g,** The phase diagram of bismuthene films with different energy gaps and band topology.

edge Dirac cones. The system is equivalent to a topologically trivial insulator with the $\mathbb{Z}_2$ topological invariant $\nu = 0$. On the other hand, an energy gap can also be opened by reducing the substrate coupling, (*e.g.*, by pulling the substrate slightly away from the substrate, as shown in Supplementary Figure 17**a**.) In this case, the "Fermi string" ESB emanates from the valence band. Consequently, there is only one edge Dirac cone traversing the bulk band gap, indicating that the system with a weakened substrate coupling is in the 2D topological insulator phase. This is consistent with the fact that freestanding bismuthene is a 2D topological insulator. A summary of topological phases related to 2D Weyl semimetals

is shown in Supplementary Figure 17**g**.

XII. Toy Model of Fermi String Edge States

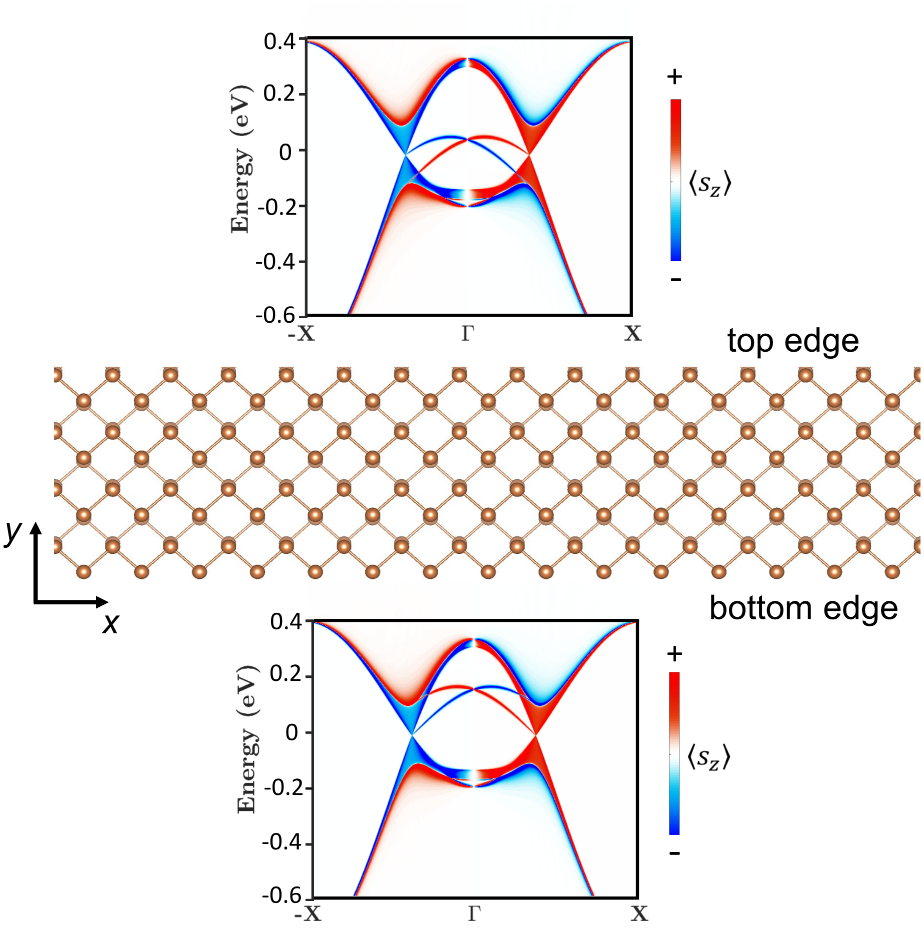

Supplementary Figure 18: Projected bulk bands and topological edge bands of a 2D Weyl-semimetal ribbon calculated with the s_z-conserved tight-binding model. The bands are colored according to the spin polarization $\langle s_z \rangle$ of the states.

We calculated the edge spectrum of a 2D Weyl-semimetal ribbon using a tight-binding model whose low-energy effective Hamiltonian is

$$\mathcal{H}^{\text{Weyl}}(\boldsymbol{k}) = \tau_z(v_x k_x \sigma_x + \Delta k_x) + v_y k_y \sigma_y + \lambda_{\text{SOC}} \tau_z \sigma_z s_z + \lambda_{\text{Dip}} \sigma_z, \tag{33}$$

where τ_i, σ_i, and s_i $(i = x, y, z)$ are Pauli matrices for valley, sublattice, and spin degree of freedom, respectively. $\mathcal{H}^{\text{Weyl}}(\boldsymbol{k})$ is a simplified version of $\mathcal{H}_{\text{eff}}$ in the main text with

$\lambda_V = \lambda'_V = 0$. In this model, s_z is conserved and thus, the spin-up and spin-down sectors are decoupled. The band gap vanishes when $\lambda_{\text{SOC}} = \lambda_{\text{Dip}}$. The gapless bulk bands and topological edge bands on the two opposite edges of the ribbon are plotted in Supplementary Figure 18. The edge spectrum from the bottom edge is similar to the DFT result shown in Figs. 4A of the main text. The in-gap edge band connects the Weyl node and the bottom of the conduction band in each spin sector. The lower parts of the "X"-shaped edge bands constitute the Fermi string band that connects the two Weyl nodes. On the top edge of the ribbon, the in-gap edge band connects the Weyl nodes and the top of the valence band in each spin sector. The connection patterns on the opposite edges follow from particle conservation.

XIII. Band Structure of Epitaxial Bismuthene in a Zeeman Field

For the 2D Weyl fermion states in Bi/SnS and Bi/SnSe, we found the spin is canted with large $\langle s_z \rangle$ component as shown in Supplementary Figure 14. Based on our calculation, the large $\langle s_z \rangle$ component remains robust even with a time-reversal-symmetry-breaking Zeeman field in the vertical direction. The only prominent effect of the Zeeman field is raising one Weyl cone and lowering the other in energy according to the spin polarization of the valley, as shown in Supplementary Figure 19. As a result, the magnetic field in the z direction can be utilized to tune the effective chemical potential of the two valleys in this system.

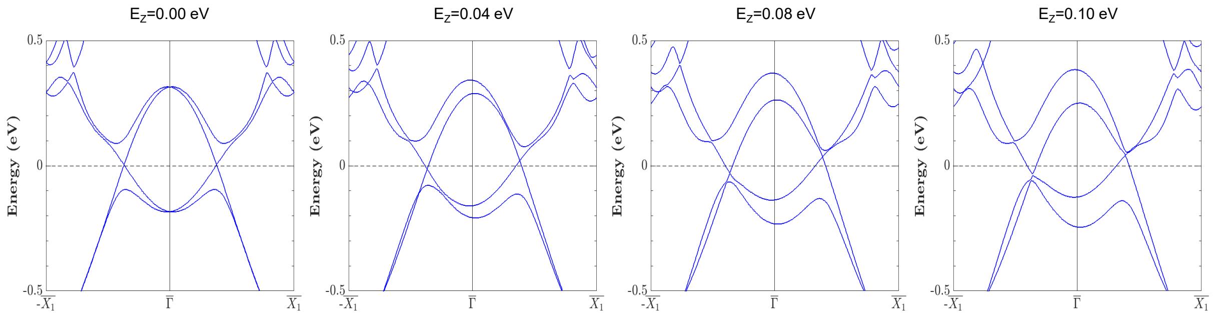

Supplementary Figure 19: Band structure of bismuthene/SnSe heterostructure in the presence of an on-site Zeeman energy, $E_Z = 0$, 0.04, 0.08, and 0.10 eV..

XIV. Exotic Properties of 2D Weyl Semimetals

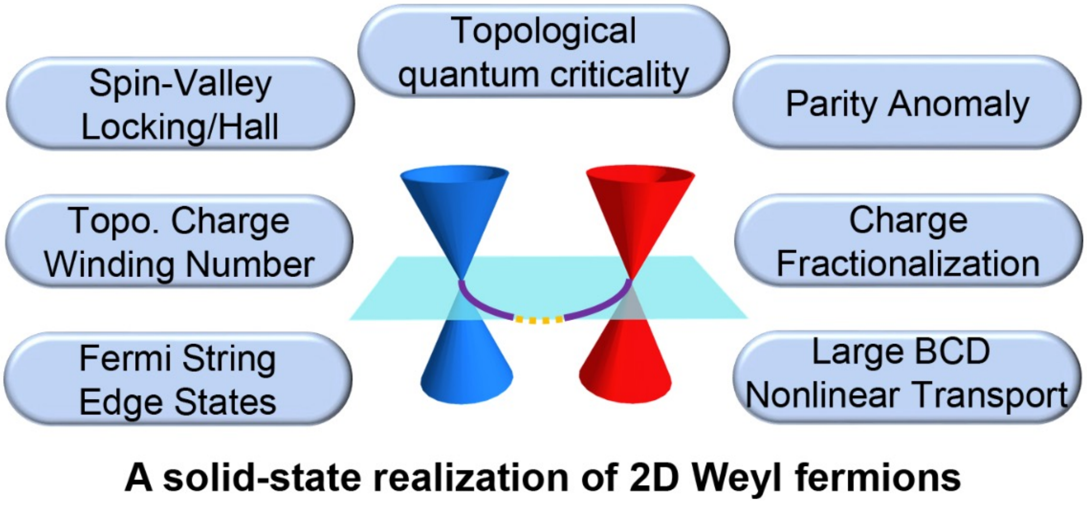

Supplementary Figure 20: Summary of exotic properties of 2D Weyl semimetals.

Supplementary References

1. R. Yu, *et al.*, *Phys. Rev. B* **84**,075119 (2011).
2. I. Sodemann, *et al.*, *Phys. Rev. Lett.* **115**,216806 (2015).
3. W.-F. Tsai, *et al.*, *Nat. Commun.* **4**,1500 (2013).
4. M. Ezawa, *et al.*, *Phys. Rev. Lett.* **110**,026603 (2013).
5. T. Kitagawa, *et al.*, *Phys. Rev. B* **84**,235108 (2011).
6. F.D.M. Haldane, *Phys. Rev. Lett.* **61**,2015 (1988).
7. G. Kresse, *et al.*, *Phys. Rev. B* **54**,11169 (1996).
8. J.P. Perdew, *et al.*, *Phys. Rev. Lett.* **77**,3865 (1996).
9. N. Marzari, and D. Vanderbilt, *Phys. Rev. B* **56**,12847 (1997).
10. I. Souza, *et al.*, *Phys. Rev. B* **65**,035109 (2001).
11. A.M. Arash, *et al.*, *Computer Physics Communications* **10**,1016 (2008).
12. C. Franchini, *et al.*, *Journal of Physics: Condensed Matter* **10**,1088 (2012).
13. C.-C. Lee, *et al.*, *Journal of Physics: Condensed Matter* **30**,295502 (2018).
14. F. Bisti, *et al.*, *Phys. Rev. X* **7**,041067 (2017).
15. W. Ku, *et al.*, *Phys. Rev. Lett.* **104**,216401 (2010).
16. S. Kourtis, *et al.*, *Phys. Rev. B* **93**,041109 (2016).
17. H. Inoue, *et al.*, *Science* **351**,1184 (2016).
